# Dynamics of rumen microbiome in sika deer (*Cervus nippon yakushimae*) from unique subtropical ecosystem in Yakushima Island, Japan

**DOI:** 10.1038/s41598-022-26050-z

**Published:** 2022-12-14

**Authors:** Misaki Eto, Tetsukazu Yahara, Arika Kuroiwa, Katsunori Shioya, Gilberto E. Flores, Natsuko Hamamura

**Affiliations:** 1 Graduate School of Systems Life Sciences, Kyushu University, 744 Motooka, Fukuoka, 819-0395 Japan; 2 Department of Biology, Faculty of Science, Kyushu University, 744 Motooka, Fukuoka, 819-0395 Japan; 3 Kyushu Natural Environmental Research Co. Ltd, 1159-5 Haramizu Kikuyoumachi, Kikuchi-Gun, Kumamoto, 869-1102 Japan; 4 Department of Biology, California State University, Northridge, 18111 Nordhoff Street, Northridge, CA 91330 USA

**Keywords:** Microbial ecology, Microbial communities

## Abstract

Yaku sika deer (*Cervus nippon yakushimae*) are endemic to Yakushima Island, whose landscape covered with primary evergreen forest is recognized as a World Heritage Site. In this study, the rumen bacterial microbiota (RBM) of wild Yaku sika was characterized using high throughput sequencing of bacterial 16S rRNA genes combined with targeted cultivation and functional analyses. Comparative analyses of RBM datasets from other ruminant animals revealed distinct community structure among domesticated and wild ruminants. Wild Yaku sika RBM exhibited higher species richness than other sika deer (i.e. wild Ezo sika and domesticated sika deer), likely reflecting their dietary variations associated with unique ecosystem in the island. The Yaku sika RBM of high deer population density samples exhibited higher diversity and contained higher proportion of *Firmicutes* than those of lower density samples. Moreover, the highest abundance of tannase gene were observed in individuals from the highest population density area, consistent with the previous observation that Yaku sika in the high density areas expanded their feed to include tannin-rich unpalatable plants. This study indicated that RBM of unique wild Yaku sika contribute to the flexibility of dietary shift and thus maintaining nutritional status of Yaku sika under high density conditions.

## Introduction

The rumen harbors highly diverse microbial communities composed of bacteria (up to 10^11^ cells/ml), methanogenic archaea (10^6^ cells/ml), fungi (10^3^–10^6^ zoospores/ml), and protozoa (10^4^–10^6^ cells/ml)^1,2^. These microbial symbionts play a critical role in herbivores allowing them to degrade plant materials into fermentable saccharides, which are then converted to volatile fatty acids providing up to 70% of the animals energy needs^3,4^. The plant material is composed of various polysaccharides including cellulose and hemicellulose, pectin, and starch. The primary polysaccharide degraders are bacteria, mainly from the genera *Ruminococcus, Fibrobacter, Butyrivibrio, Ruminobacter, Lachnospira,* and *Prevotella*^4–6^*.* Bacteria are the functionally crucial, most numerically dominant component of the rumen microbiome and some bacteria also play an important role in the detoxification of plant secondary compounds (PSCs)^7–9^. Among PSCs, tannin is widely distributed in plants^10,11^, and the second most abundant group of plant phenolics after lignins^12^. The intake of high doses of tannin can limit nutrient availability to animals by binding to proteins and other macromolecules resulting in weight loss^12,13^, and damage to the kidneys, liver and gastrointestinal tract^13,14^. Herbivores may adapt to the toxic effect of tannin by harboring symbiotic bacteria along their digestive tract capable of degrading hydrolysable tannins using tannase enzymes^15^. Some tannin-degrading bacteria have been isolated from the rumen of goats (*Streptococcus* spp.)^16^ and East African ruminants (*Streptococcus, Butyrivibrio,* and *Selenomonas* spp.)^17^.

Although the symbiotic microorganisms in the rumen are difficult to characterize by traditional cultivation-dependent approaches, recent advances in cultivation-independent techniques based on next-generation DNA sequencing allow us to infer the structure–function relationships of rumen bacterial microbiota (RBM). To date, the majority of RBM studies have focused on domesticated ruminants such as goats and dairy cows^18,19^, while reports on wild ruminants are rather limited to a few species including Norway reindeer^20^, boreal cervids^21^, and Canadian cervids^22^ which live in subfrigid or frigid zones. From these and other studies, diet type and host species are among the key factors that shape the rumen microbiota^23,24^, and the diet variety was shown to associate with species richness in gut microbiome^25^. Thus, wild ruminants associated with natural habitats and wide diet varieties are likely to have distinct and more diverse RBM compared to domesticated ruminants.

Japanese sika deer (*Cervus nippon*) are widely distributed on the main islands of Japan where they feed on a diversity of plants available in the subalpine coniferous forests of Hokkaido (*C.n. yesoensis*) in the north, to the warm temperate evergreen broad-leaved forests of Kyushu (*C.n. nippon*) in the south. Yaku sika deer (*C.n. yakushimae*) are a subspecies of Japanese sika deer and are endemic to Yakushima Island, which is located 70 km south of Kyushu, Japan (30°20'N, 131°30'E). The mountainous area of Yakushima Island was registered with the World Nature Heritage List in 1993 for its unique landscape covered with primary evergreen forest where conifers and broad-leaved trees coexist from the coast to the summit of 1936 m, which is a home to some 1,900 species and subspecies of vascular plants^26^. Coniferous forests are observed at higher elevation (> 900 m), and broad-leaved forests dominated by Fagaceae are observed at lower elevation (< 900 m). On Yakushima Island, wild *C. n. yakushimae* graze on a range of food including woody plants, vines, herbaceous plants, ferns, moss, and fungi^27^. Recent study by Higashi et al.^28^ determined DNA sequences of plant taxa contained in feces of Yaku sika collected in different seasons (i.e. April, June, August, and October), and showed that Yaku sika were selectively foraging on some small plants, including ferns, shrubs, herbs, and bryophytes. In addition, the evergreen tall trees including Fagaceae and Lauraceae were also detected in deer feces across all seasons^28,29^.

**Figure 1:**
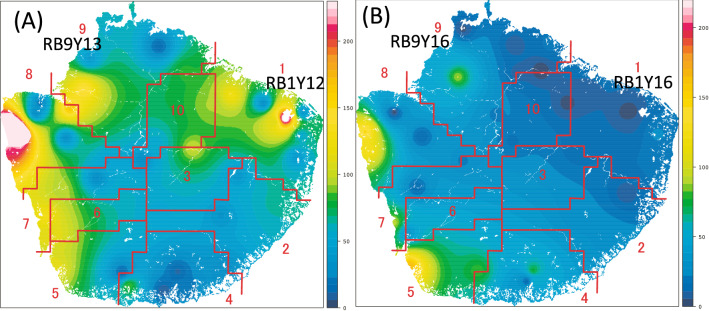
A contour map of estimate Yaku sika population density in 2013 (**A**) and 2016 (**B**). The numbers on the maps indicate the IDs of the ten areas separated by river boundaries (RB). Samples used in this study were obtained from following areas on the map; RB9Y13, RB9Y16, RB1Y12, and RB1Y16. Color keys show the estimated deer density as the number of deer individuals per km^2^. Redrawn from figures in Kagoshima Prefecture Nature Conservation Division^33^ under permission of Kagoshima Prefecture.

The Yaku sika deer population on Yakushima Island decreased dramatically in the 1950’s due to overhunting. More recently, however, the population has recovered under protection (i.e. from 24 to 95–112 heads per km^2^ in 1995 and 2007, respectively)^30,31^. This rapid and large population increase caused serious damage to the natural forest ecosystem^32^. To mitigate further environmental impacts, a large number of deer were culled in 2012 and 2013 (3,811 and 4,556, respectively)^33^. By 2016, the estimated deer population decreased significantly in eastern areas of the island, shown as the river boundary (RB) area 1 in Fig. 1 (RB1Y16), while population density was still relatively high in other regions including the northwestern (RB area 9, RB9Y16, in Fig. 1), western, and southern parts of the island^33^. The deer in these high density areas have expanded their feed to include tannin-rich unpalatable plants, fallen leaves, and woody material to meet their nutritional needs^28,34^. Wild Ezo sika deer (*C.n. yesonensis*) inhabiting Hokkaido, the northern island of Japan, were observed to feed on accessible woody materials containing high level of tannin during the winter season, and it was suggested that Firmicutes, the dominant phylum of fungi, and the tannin-degrading rumen bacteria play an important role in survival of sika deer under limited feed condition^35,36^. Consequently, we hypothesized that the dietary shifts of wild Yaku sika deer in high density areas have been accompanied by changes in the RBM that includes more tannin-degrading bacteria which allows these deer to adapt to feeding on tannin-rich diet.

To test this hypothesis, we characterized the rumen bacterial microbiota of distinct populations of wild Japanese Yaku sika deer associated with the unique evergreen forest ecosystem of Yakushima Island. The rumen bacterial microbiota of the wild Yaku sika deer was first compared with those from previous studies of other wild and domesticated ruminants^23,37^. Furthermore, regional and temporal variation of the rumen microbiome among deer from areas with different population densities (as a proxy for consumption of tannin-rich plants) were investigated using both cultivation-dependent and independent approaches.

## Results and discussion

### Rumen microbiota of wild Yaku sika and other ruminants

**Figure 2:**
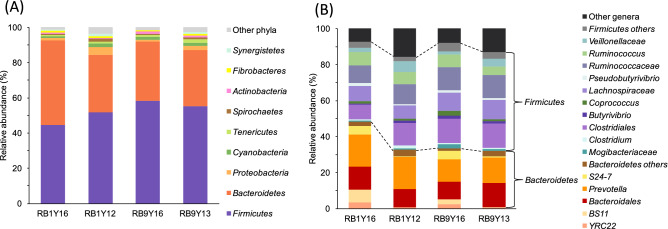
Yaku sika rumen bacterial community compositions at phylum level (**A**) and genus level (**B**). “Other phyla” contains several phyla with < 1% relative abundance. “Other genera” contains several genera among other phyla.

The rumen bacterial microbiota (RBM) of the wild Yaku sika deer was characterized using amplicon sequencing of bacterial 16S rRNA genes collected from 19 culled deer in 2012 (n = 4), 2013 (n = 5), and 2016 (n = 10) (Table 1). A total of 632,383 high-quality sequences used for analysis resulted in 194,802 OTUs based on the 97% sequence identity, and 433,358 of those sequences were assigned to 81,981 OTUs and classified, at least, to the phylum level, while the rest of the OTUs could not be classified using Greengenes database^38^. The RBMs of the wild Yaku sika deer were assigned to 22 phyla and 113 families, and *Firmicutes* and *Bacteroidetes* were the dominant phyla accounting for 89.2 ± 4.1% of the assigned sequences (Fig. 2A). Those two phyla consisted of 29 families, including *Ruminococcaceae, Lachnospiracea, Prevotellaceae,* and *Paraprevotellacea,* which are all known rumen-inhabiting bacterial groups^23,39^*.* At the genus level, *Prevotella* spp. were the most abundant bacteria and comprised 15.5 ± 6.7%, followed by *Ruminococcus,* and unclassified bacteria in *Clostridiales, Bacteroideles, Ruminococcaceae,* and *Lachnospiracea* (Fig. 2B). These top six bacterial groups comprised 68.3 ± 6.6% of the classified sequences from Yaku sika RBMs and were also detected as the most abundant groups in a large selection of other ruminants^23^, suggesting that these taxa comprise the “core bacterial microbiome” of ruminants^40^.

**Table 1:** Sample information and sequence summary.

Group name	RB area	Year	Location	Deer density (head km^-2^)	Sample name	Season^c^	Age	Weight (kg)	Sex	Raw reads	Paired reads	Paired reads without chimera
RB1Y16	1	2016	Koseda	10.5^a^	K1	Spring	3+	30	M	252,170×2	79,059	49,935
			Koseda	10.5^a^	K2	Spring	3+	30	M	356,860×2	99,674	64,546
			Koseda	10.5^a^	K3	Spring	3+	25	M	237,777×2	79,923	50,559
			Nagamine	10.5^a^	N1	Spring	3+	32	F	244,715×2	87,716	54,202
			Nagamine	10.5^a^	N2	Spring	3	32	F	308,716×2	109,020	71,259
RB1Y12	1	2012	Koseda	93.5^b^	K4	Summer	1	10	F	64,342×2	3,805	2,183
			Koseda	93.5^b^	K6	Summer	1	13	M	153,770×2	10,548	6,051
			Koseda	93.5^b^	K7	Summer	2	18	F	103,722×2	5,013	2,655
			Koseda	93.5^b^	K8	Summer	1	10	F	66,888×2	9,149	2,151
RB9Y16	9	2016	Nagata	51.6^a^	Nt1	Spring	3	25	M	281,776×2	109,460	61,864
			Yoshida	51.6^a^	Y1	Spring	3	21	F	216,360×2	72,959	49,600
			Isso	51.6^a^	I1	Spring	3	25	F	230,468×2	71,146	42,188
			Miyanoura	51.6^a^	M1	Spring	3	32	M	175,452×2	55,643	32,938
			Miyanoura	51.6^a^	M2	Spring	3	31	F	168,005×2	58,435	38,791
RB9Y13	9	2013	Miyanoura	69.8^b^	M3	Summer	3+	24	F	261,039×2	91,346	57,989
			Miyanoura	69.8^b^	M4	Summer	1	17	M	174,070×2	59,635	35,303
			Miyanoura	69.8^b^	M5	Summer	1	12	F	56,028×2	3,277	1,908
			Miyanoura	69.8^b^	M6	Summer	1	11	M	109,556×2	6,186	3,789
			Miyanoura	69.8^b^	M7	Summer	1	15	M	150,153×2	7,771	4,472

^a^ Deer density was calculated as an average within the river boundary area^33^.

^b^ Deer density was calculated as an average within the location^34^.

^c^ Spring: April. Summer: June, July, and August.

**Figure 3:**
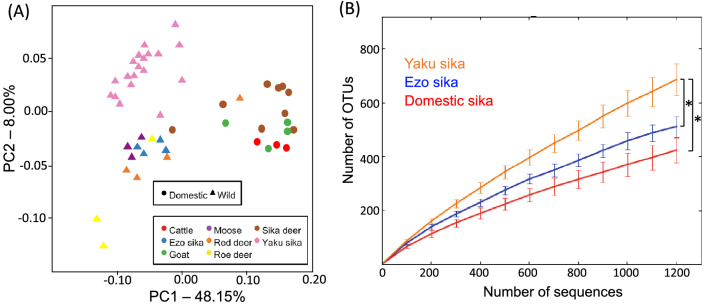
(**A**) PCoA based on weighted UniFrac distance among ruminants. The weighted UniFrac distance was tested using permutational multivariate analysis of variance. (**B**) Rarefaction curves of 16S rRNA OTUs among sika deer. The number of OTUs of Yaku sika rumen bacterial microbiota was compared to those of Ezo sika and domestic sika deer. *P ≤ 0.001.

The RBM of the wild Yaku sika deer was compared with those of other wild and domesticated ruminants characterized in previous studies^23,37^. Principal coordinates analysis (PCoA) based on weighted UniFrac distance showed that the community structure among domestic and wild ruminants were significantly different (Fig. 3A; PERMANOVA, pseudo-*F* = 29.044, *P* = 0.001; PERMDISP, *F* = 1.100, *P* = 0.29). This result indicates that RBM of wild ruminants grazing on a variety of forage types are compositionally different than those from domestic ruminants consuming feed rich in starch and protein, consistent with the previous report that diet is a major factor driving the composition of microbiota^41,42^. Among three sika deer groups, Yaku sika (*C.n. yakushimae*)*,* domestic sika deer (*C.n. nippon* ), and wild Ezo sika (*C.n. yesoensis),* species richness is significantly higher in RBM of Yaku sika (2,145.5 ± 225.2; mean ± SD) than those of Ezo sika (1,242.3 ± 127.6) and domestic sika deer (1,112.1 ± 150.5) (Fig. 3B, one-way ANOVA, F = 120.93, *P* ≤ 0.001; Tukey–Kramer test, *P* ≤ 0.001). Similarly, the Shannon–Wiener index was also significantly higher in Yaku sika than other two sika groups (Yaku sika: 9.63 ± 0.48, Ezo sika: 8.55 ± 0.21, domestic sika deer: 7.61 ± 0.60; Tukey–Kramer test, *P* = 0.003 and *P* ≤ 0.001, respectively), and that of wild Ezo sika was higher than domestic sika deer (Tukey–Kramer test, *P* = 0.017) consisting with the higher RBM diversity among wild ruminants. The wild Ezo sika inhabit the cool temperate and sub-alpine/alpine mixed forests in Hokkaido^43^, and samples for RBM analysis were collected during winter when Ezo sika’s home ranges are characterized with lower snow cover, high availability of bamboo grass and coniferous covers^23,44^. Consequently, the high diversity in Yaku sika RBM is likely associated with high diversity in plant species available in the warm-temperate area of Yakushima Island, which then possibly contribute to the flexibility of their diets posed under high population density conditions^34^.

### Dynamics of Yaku sika RBM

The regional and temporal variations of RBM among Yaku sika were examined in two regions (region 1 and 9 in Fig. 1) with distinct deer population density trends in Yakushima Island. Deer population in region 1 was dramatically decreased by 2016 due to high hunting pressure since 2012 (from 93.5 to 10.5 heads per km^2^ in 2012 and 2016, respectively; Table 1), while region 9 maintained higher deer population (69.8 and 51.6 heads per km^2^ in 2013 and 2016, respectively)^34^. Deer examined in this study were obtained from region 1 in 2016 (RB1Y16; n = 5) and 2012 (RB1Y12; n = 4), and from region 9 in 2016 (RB9Y16; n = 5) and 2013 (RB9Y13; n = 5) (Table 1). The RBM taxonomic composition were compared among those four sample groups (Fig. 2), and the relative abundances of *Firmicutes, Bacteroidetes* and *Proteobacteria* phyla were significantly different among the groups (Kruskal–Wallis, *P*_*Bonferroni*_ ≤ 0.05). Between the dominant *Firmicutes* and *Bacteroidetes* phyla, *Bacteroidetes* was significantly enriched in the lowest deer density sample RB1Y16 (51.26 ± 4.00%; one-way ANOVA, *F* = 6.082, *P* = 0.006; Tukey–Kramer test, *P* ≤ 0.001), while abundance of *Firmicutes* was the lowest (40.70 ± 3.24%) compared with other samples with higher deer population densities (i.e. RB1Y13, RB9Y16, RB9Y12; Fig. 2A and Fig. S2). The degradation of a high fiber diet was reported to correlate with *Firmicutes* abundance, which enables efficient energy extraction from high fiber diets, while the degradation of plant starches, proteins, and fats was associated with the abundance of *Bacteroidetes* in rumen^45,46^. Our results showing the shift in *Firmicutes-*dominant to *Bacteroidetes-*dominant population in region 1 (RB1Y16) are consistent with the shift in diet from fiber rich forage to more nutritious starch and fat rich diet as population density decreased in region 1 after the cull in 2016^34^. In contrast, Yaku sika RBM from high population density groups (i.e. RB1Y12, RB9Y13 and RB9Y16) containing dominant *Firmicutes* would be better adapted to the fiber rich forage^36,47,48^.

**Figure 4:**
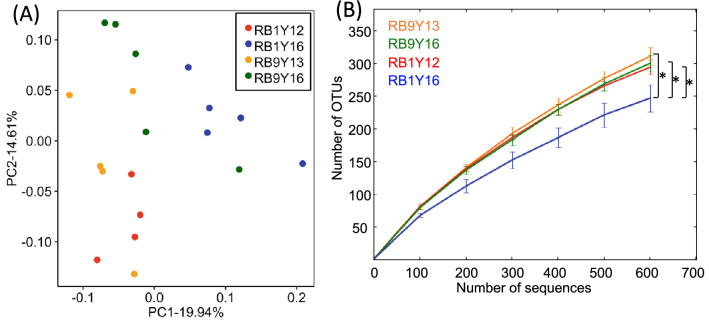
(**A**) PCoA analysis based on weighted UniFrac distance and (**B**) rarefaction curves of 16S rRNA OTUs among Yaku sika groups. *P ≤ 0.015.

Furthermore, deer from high population density areas hosted compositionally distinct RBMs compared with those from lowest population density RB1Y16 group (Fig. 4A and Table S3, PERMANOVA,* P* ≤ 0.03 ; PERMDISP, *F* = 0.379, *P* = 0.784). The comparison between before and after the cull in two areas (RB1 and RB9) showed significant difference only in region 1 (RB1Y12 vs. RB1Y16, R^2^ = 0.292, *P* = 0.007; RB9Y13 vs. RB9Y16, R^2^ = 0.157, *P* = 0.059), where population density decreased dramatically after the cull by 2016. The RBMs from deer collected in spring of 2016 with similar age were significantly different (RB9Y16 vs. RB1Y16, R^2^ = 0.209, *P* = 0.032) based on the area of sampling, suggesting that these differences are in part driven by deer density. In addition, the RBMs from high population density areas showed higher species richness compared with those from the lowest population density RB1Y16 samples (Fig. 4B; Tukey–Kramer test, *P* ≤ 0.02). In general, species-rich communities are resilient against changes in resource composition and maintain their function in case of environmental changes^49^. For instance, a previous study in woodrats showed an increase in microbiome diversity associated with intake of PSCs, which is considered to be the result of adaptation to plant toxins^50^. Recent studies showed that Yaku sika selectively forages small plants in understory vegetation, if they are available, and also utilize evergreen tall trees^28,29^. Among the evergreen trees, Fagaceae and Lauraceae (in specific, *Machilus* spp.) are the predominant food sources for Yaku sika in lower elevation areas including area 1 and 9 in this study (< 900 m), and the leaves from these trees are known to contain high dose of tannin^51–53^. In low deer density areas, the availability of the small plants is higher and more species-rich understory vegetation is found, thus the dependence of Yaku sika on food derived from tall trees is lower in those areas^27^. The deer in high density areas have expanded their feed to include tannin-rich unpalatable plants, fallen leaves^27,54^, and woody material to meet their nutritional needs^28,34^. Thus, the higher diversity of RBM in Yaku sika under high population density may enable deer to scavenge a wider variety of plants and tolerate plant toxins, thus maintain nutritional status even under high density conditions^29,55^.

### Tannin-degrading bacteria in Yaku sika RBM

In order to investigate the contribution of Yaku sika RBM in detoxification of PSCs, tannin-degrading bacteria were examined by cultivation-dependent and independent approaches. This is the first attempt to isolate tannin-degrading bacteria from Yaku sika rumen contents, and a total of 12 colonies representing 4 species were successfully isolated (Table 2). Majority of the isolates belonged to a known tannin-degrading genus *Streptococcus* spp., which is also a known rumen-inhabiting bacterial group^56^. *Streptococcus gallolyticus* subsp. *gallolyticus* is known to produce a cytoplasmic tannase (TanB) as well as an extracellular tannase (TanA), and are suggested to play an important role in digestion of tannin rich diets^57^. In addition, other isolates closely related to *Escherichia coli* strain WTPii24 and *Pseudomonas* sp. strain 2C were obtained (Table 2). Previously, tannin-degrading *E. coli* and *Pseudomonas* strains were isolated from feces of desert wood rats^15^, the rumen of white-tailed deer^58^, and from soil^59^.

**Table 2:** 16S rRNA gene sequences of tannin-degrading bacteria isolated from Yaku sika rumen.

Isolate	Closest cultured strains (Accession number)	Sequence identity (%)	Number of isolates
S7C8	*Pseudomonas veronii* strain CIP104663 (NR_028706)	99	2
S7C10	*Streptococcus gallolyticus* strain YR2 (KJ765727)	99	8
S7C12	*Streptococcus infantarius* strain C12 (KY801940)	99	1
S2C15	*Escherichia coli* strain WTPii241 (MH396737)	99	1

Presence of tannin-degrading bacteria in Yaku sika RBM was also examined by a PCR-based approach. Two sets of PCR primers were designed based on *tanB* sequences of known tannin-degrading bacteria (Fig. S1, Table S1). Primer specificity and PCR condition were confirmed using DNA from *S. gallolyticus* and *P. stutzeri* as positive controls. PCR with rumen DNA (two samples from RB9Y16) was also performed to verify these primer sets, and a positive amplification was observed only with the group1 primer set targeting *tanB* from *Streptococcus* and *Lactobacillus* (Fig. S1). The amplified tannase sequences from Yaku sika rumen contents were confirmed to be *tanB* genes closely related to that of *Streptococcus gallolyticus* UCN34 (amino acid sequence identity 94–96%) (Fig. S3). Thus, further *tanB* quantification was conducted using the group1 primer set.

**Figure 5:**
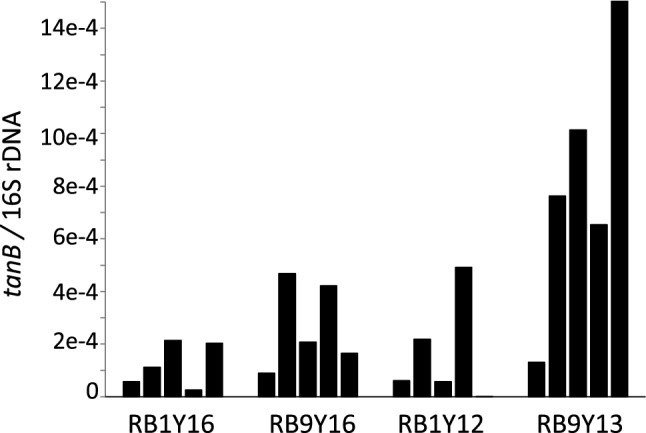
The ratios of *tanB* to16S rRNA genes copy numbers (*tanB/*16S rDNA). Bars indicate the mean values of triplicate qPCR replicates from individual deer (n = 5/group), and error bars representing the standard deviation are shown but absent due to their small scale.

To gain insight regarding the relationship between the relative abundance of the tannase-possessing bacterial population in RBM and Yaku sika deer population density, quantitative PCR was performed to determine the ratios of *tanB* to 16S rRNA gene copy numbers (*tanB*/16S rDNA). The results showed that, although there was high individual variation within groups, the highest abundance of *tanB* genes were observed in individuals from areas with the highest population density (i.e. RB9Y13), while lower *tanB* abundances were observed among individuals from areas with the lowest deer density (i.e. RB1Y16; Fig. 5). This result also supports the previous observation that Yaku sika from high density areas consume tannin-rich unpalatable plants, fallen leaves^27,54^, and woody materials more frequently than those from low density areas to meet their nutritional needs^28,34^. Similar to previously reported herbivores capable of expanding the niche range of their diet by harboring PSC-degrading bacteria in their digestive systems^50,60^, Yaku sika may also have adapted to utilize tannin-rich plants under high population pressure by harboring tannin-degrading bacteria in their RBM.

## Conclusions

This is the first study to characterize the RBM of wild endemic species of Yaku sika in Yakushima Island. Yaku sika exhibited more diverse RBM compared to Ezo sika and domestic sika deer, likely reflecting their dietary variations associated with their unique ecosystem under the warm temperate climate of the island. Among Yaku sika RBM, significantly higher diversity and distinct community structure were observed from high population density samples (i.e. RB1Y12, RB9Y13 and RB9Y16) compared to those from lower density samples (RB1Y16) supporting our hypothesis. Moreover, tannin-degrading bacteria were detected from Yaku sika rumen by both cultivation-dependent and independent approaches. Yaku sika RBM are likely contributing to flexibility of dietary shifts including tannin-rich plants consumption and to maintain nutritional status of Yaku sika under high population density conditions.

## Materials and methods

### Ethics statement

Based on the Yaku sika Specific Management Plan (https://www.pref.kagoshima.jp/ad04/sangyo-rodo/rinsui/shinrin/syuryo/documents/58352_20170330170915-1.pdf), the Forestry Agency and the Hunting Association culled Yaku sika deer under the permission of Kagoshima Prefecture regarding the extermination of harmful birds and beasts based on the Wildlife Conservation Law*.* Rumen samples from wild Yaku sika deer were obtained from animals culled by licensed hunters for this Management Plan. The harvested deer were immediately transported to a licensed abattoir (Miyanoura, Yakushima-cho, Kagoshima) where rumen samples were collected. The hunting was done according to local legislation and no animals were killed specifically for this study.

### Sampling of rumen contents from Yaku sika

A total of 19 culled deer captured in the summer of 2012, 2013, and the spring of 2016 were examined (Table 1). Previous work examined the plant species compositions in stomach contents of Yaku sika, and it was shown that, the occurrence of major plant species in deer diets were not significantly different between seasons (i.e. summer and winter)^29^. Total rumen contents were collected into a 50-ml sterile tubes using sterile spoons and immediately placed on ice until transported back to the lab (within 5 days) where they were stored at − 80 °C. The rumen contents were freeze-dried (VD250-R; TAITEC Co., Saitama, Japan), homogenized in a coffee grinder (Russell Hobbs, Failsworth, UK) which was thoroughly washed and wiped with 70% ethanol after processing each sample, and stored at − 80 °C for molecular analysis.

### Molecular analysis

DNA was extracted from each rumen sample of about 2–3 g using PowerMax Soil DNA Isolation Kit (QIAGEN, Hilden, Germany) following the manufacturer’s instructions. DNA concentration was determined using Nanodrop 2000c (Thermo Fisher Scientific, Waltham, MA, USA). The V1–V3 regions of bacterial 16S rRNA genes were amplified by first PCR using primers Ba515Rmod1 and Ba9F^23^. After first PCR, products were purified using Agencourt AMpure XP A63880 (Beckman Coulter Inc., California, USA), and second PCR were conducted using primers containing appropriate Miseq adapters for sequencing. The purified amplicons were sequenced on Illumina MiSeq using v3 (300 bp paired end) sequencing kit. Sequence data was deposited in DDBJ database under accession number DRA007953.

Sequences were analyzed using QIIME 1.9.0^61^. Paired reads from MiSeq reporter were merged with 15% allowable mismatch bases with at least 6 overlapping bases. The primer sequences were searched by allowing 20% error and removed using cutadapt 1.12. Reads in which the primer sequences were not identified or sequences shorter than 200 bases were discarded. Finally, reads containing 80% or more of bases with quality score above 20 were extracted.

Chimeric reads were removed from the pre-processed fasta file using usearch v6.1^62^. Operational taxonomic units (OTU) were defined by clustering reads at 97% sequence identity using QIIME 1.9.0^61^. Taxonomy was assigned to representative sequences from each OTU using Greengenes v12_10^38^, and the number of reads from each OTU were tabulated. Unclassified OTUs were discarded, and the relative abundance was tabulated at each taxonomic level. The remaining sequences were rarefied to 1,200 sequences per sample, and used to calculate two alpha-diversity metrics (observed OTUs and Shannon–Wiener index). To compare yaku sika rumen bacterial communities, low abundance OTUs were removed (< 0.005%), sequences were aligned using PyNAST^63^, and a phylogenetic tree was estimated using FastTree 2.1.3^64^. The phylogenetic tree was used to calculate UniFrac distances to compare rumen microbial communities. Principal Coordinates Analysis (PCoA) was performed using weighted UniFrac distances and visualized using the R 3.3.2^65^.

To compare the microbiome of wild Yaku sika to other wild and domesticated ruminants, 16S rRNA gene amplicon data from the SRA (Sequence Read Archive, www.ncbi.nlm.nih.gov/sra) were downloaded (Table S1), processed identical as describe above, and rarefied 1,200 sequences per sample were used for further analyses.

### Statistical analysis

Statistical analyses were performed using R v3.3.2^65^. We first determined if data were normally distributed using the Shapiro–Wilk test. When normally distributed, relative abundance or diversity index were tested using one-way analysis of variance (ANOVA) (parametric) with verification for homogeneity of variance by Bartlett test. To identify pairwise differences, multiple comparisons were conducted by Tukey–Kramer test. When not-normally distributed, the Kruskal–Wallis one-way analysis of variance (nonparametric) was used with verification for homogeneity of variance by Fligner-Killeen test. For pairwise comparisons following the Kruskal–Wallis test, we used the Steel–Dwass test. Permutational multivariate analysis of variance (PERMANOVA) and Adonis implementation were used to test for difference in community composition measured as weighted UniFrac distances (999 permutations). Permutational analysis of multivariate dispersions (PERMDISP; 999 permutations) was used to test for difference in dispersion between groups.

### Isolation of tannin-degrading bacteria

Isolation of tannin-degrading bacteria was performed as previously described by Osawa^66^ with some modifications. Briefly, 5 ml of filter sterilized (pore sizes of 0.2 µm) 2% tannic acid solution (Kanto Chemical Co., Inc., Tokyo, Japan) was overlaid onto BHI agar plates (Oxoid Ltd., Hampshire, UK) containing 1% (w/vol) agarose and 0.5% (w/vol) yeast extract. After standing for 40 min, the solution was removed and plates were rinsed three times with sterile 0.25 × Ringer Solution (Sodium chloride 2.25 g, Potassium chloride 0.105 g, Calcium chloride 6H_2_O 0.12 g, and Sodium bicarbonate 0.05 g per liter). The tannic acid-treated plates became opaque due to the formation of tannic acid and protein complex on the plate surface. For the inoculum, approximately 0.5 g rumen contents was suspended in 1 ml of Ringer Solution (1x), diluted 10 times, and 100 µl was plated on above prepared tannic acid-treated plates. Inoculated plates were anaerobically incubated at 37 °C using AnaeroPack-Anaero (Mitsubishi Gas Chemical Inc, Tokyo, Japan). Isolated colonies which formed zones of clearing on the 2% tannic acid-treated were identified as tannin-degrading bacteria. 16S rRNA gene sequences of those isolates were determined as described previously^67^ and have been deposited under accession numbers MW931767 to MW9317700.

### Tannase gene targeted primers

Tannase gene (*tanB*) sequences of tannin-degrading bacteria isolated from rumen or intestine were obtained from GenBank^68^ or Integrated Microbial Genomes & Microbiomes^69^. Two primer sets were designed to target phylogenetically distinct groups (Table S2 and Fig. S1) based on the phylogenetic analyses shown in Fig. S1; group 1 (*Streptococcus geopolitics* ATCC 43143, *S. geopolitics* JCM 7895, *Lactobacillus plantarum* JCM 1149, *L. pentosus* KB 232, *L. paraplantarum* DMS10667) and group 2 (*Pseudomonas stutzeri* CGMCC 1.1803, *P. stutzeri* JCM 5965, *Clostridium beijerinckii* DSM 53, *Burkholderia cepacia* VC13476, *Klebsiella pneumoniae* 342). To verify those primer sets, PCR was carried out with annealing temperature shown in Table S2 using rumen DNAs from high density deer population area (i.e. RB9Y13) and from tannin-degrading type strains, *Streptococcus geopolitics* JCM 7895 and *P. stutzeri* JCM 5965, as positive controls. After gel electrophoresis (1% agarose), PCR products of the expected size (177 bp and 700 bp for group1 and group2 primers, respectively) were excised from the gel and purified using QIAquick Gel Extraction Kit (QIAGEN, Hilden, Germany). Purified PCR products were cloned using the TOPO TA Cloning Kit (Invitrogen, California, USA) and sequenced. Tannase gene sequences have been deposited under accession numbers MW981287 to MW981290.

### Quantitative PCR of tannase gene and 16S rRNA genes

Quantitative PCR was performed to quantify the copy numbers of tannase genes using primers for group 1 (Tan1f and Tan1r, Table S2) with the following conditions: initial denaturation at 98 °C for 2 min, and 38 cycles of 95 °C for 3 s and 65 °C for 3 s, followed by melting curve detection. In addition, qPCR was conducted to quantify the copy numbers of 16S rRNA genes using Bac1369F and Prok1492R^70^ with following condition: initial denaturation at 98 °C for 2 min, and 32 cycles of 95 °C for 3 s and 56 °C for 3 s, followed by melting curve detection. All qPCR was carried out in triplicate by CFX Connect Real-Time PCR Detection System (Bio-Rad Laboratories*,* California, USA*)* using SsoFast ™ EvaGreen SuperMix (Bio-Rad Laboratories). For the quantitative enumeration based on the gene copy number, standard curves were prepared from a ten-fold serial dilution between 1.0 × 10^2^ and 1.0 × 10^8^ copies per reaction of the PCR amplicon generated using the M13 primers from a plasmid (pCR 4.0; Invitrogen) containing the target bacterial 16S rRNA gene or *tanB* gene sequences.

## Supplementary Information

Supplementary Information.

## Data Availability

The dataset generated during the current study are available in DDBJ database under accession number DRA007953 and in the GenBank database under accession numbers MW981287 to MW981290.
