# Supplementary material for: Dynamics of rumen microbiome in sika deer (Cervus nippon yakushimae) from unique subtropical ecosystem in Yakushima Island, Japan
Source: Sci Rep. 2022 Dec 14;12:21623. doi: 10.1038/s41598-022-26050-z (PMC9751099; doi:10.1038/s41598-022-26050-z)
Supplement: Supplementary file 1 — Supplementary Information. [file 41598_2022_26050_MOESM1_ESM.pdf]

Supplemental Information

Figure S1. Phylogenetic tree based on amino acid sequences of tannase (TanB) used for primer design. Neighbor-joining method was used and boot strap values (1000 replicates) are shown next to the branches. Bar=0.20 substitutions per sequence position.

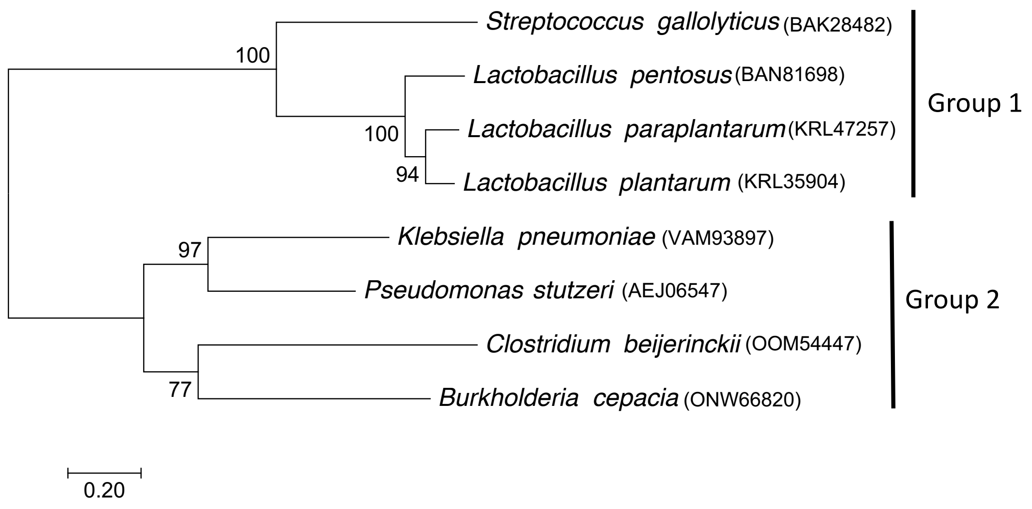

Figure S2. Relative abundance of *Firmicutes* and *Bacteroidetes* comparison among Yaku sika. The statistical analyses were conducted by using the one-way ANOVA and Tukey-Kramer test.

Group	*Bacteroidetes*	*Firmicutes*	Other phyla
RB9 Y13	41.1	47.3	11.6
	33.2	54.0	12.8
	31.7	57.9	10.4
	28.5	56.5	15.0
	34.0	46.9	19.1
RB9 Y16	34.6	55.2	10.3
	45.1	48.8	6.1
	38.7	52.0	9.2
	36.3	56.5	7.2
	30.5	60.9	8.6
RB1 Y12	34.2	52.3	13.5
	38.3	38.3	23.3
	34.7	50.7	14.6
	31.2	52.6	16.2
RB1 Y16	46.9	45.0	8.1
	52.6	39.9	7.5
	55.2	36.0	8.8
	47.1	41.2	11.7
	54.5	41.4	4.2

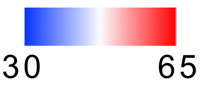

Figure S3. Phylogenetic tree based on amino acid sequences of tannase (TanB) obtained from rumen samples. Neighbor-joining method was used and boot strap values (1000 replicates) are shown next to the branches. Bar=2.0 substitutions per sequence position.

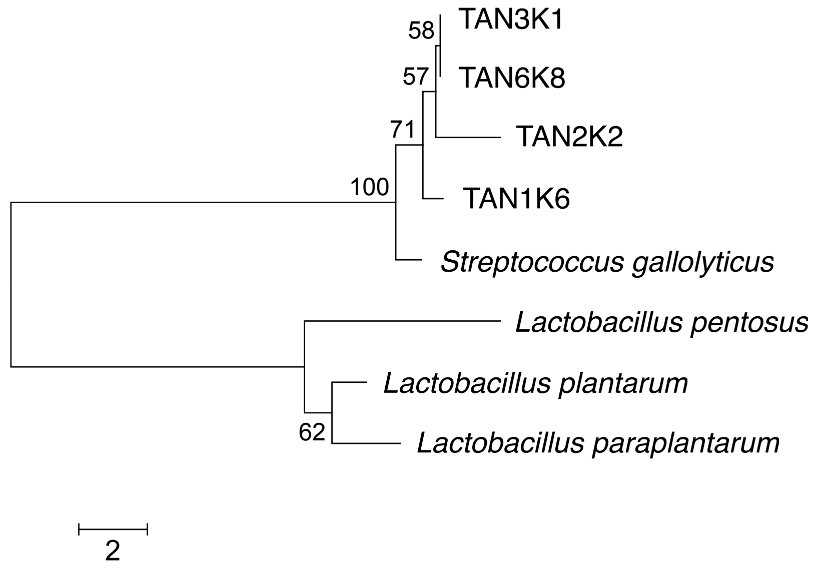

Table S1. 16S rRNA gene amplicon data from other ruminants obtained from SRA (Sequence Read Archive database).

Host	Bioproject ID	SRA ID	Reference
Cattle	PRJNA272135	SRR1774413, SRR1774419, SRR1774422	[1,2]
Ezo sika (*Cervus nippon yesoensis*)		SRR1774615, SRR1774617, SRR1774619, SRR1774622	
Goat		SRR1774338, SRR1774341, SRR1774344, SRR1774347	
Moose		SRR1775997, SRR1776000, SRR1776003	
Red deer		SRR1775181, SRR1775196, SRR1776380, SRR1776383	
Roe deer		SRR1775229, SRR 1775232, SRR1775235	
Ezo sika (*Cervus nippon yesoensis*)		SRR1774615, SRR1774617, SRR1774619, SRR1774622	
Domestic Sika deer (*Cervus nippon)*	PRJNA229770	SRR1043042 ~ SRR1043059	[3]

1. Rius, A. G. *et al.* Nitrogen metabolism and rumen microbial enumeration in lactating cows with divergent residual feed intake fed high-digestibility pasture. *J Dairy Sci.* **95**, 5024-5034, (2012).
2. Henderson, G. *et al.* Rumen microbial community composition varies with diet and host, but a core microbiome is found across a wide geographical range. *Sci. Rep.* **5**, 14567, (2015).
3. Li, Z. *et al.* Bacterial community composition and fermentation patterns in the rumen of sika deer (*Cervus nippon*) fed three different diets. *Microbial Ecol.* **69**, 307-318, (2015).

Table S2. Tannase targeted primers used in this study.

	Primer	Sequence (5'-3')	Product size	Annealing Temperature
group1	Tan1f<br>Tan1r	GGAACGAGTGCTGGBGGTG<br>CCAYTCATAAGCCATRTCWGCGTG	177bp	70° C
group2	Tan2f<br>Tan2r	TATTTCATBGGMKGCTCKGACGG<br>CCGTGCCASAGRATCAACTTGCC	700bp	69° C

Table S3. Adonis (Permanova; 999 permutations) results for community compositions between Yaku sika sample groups based on weighted UniFrac distances.

	d.f.	*SS*	*MS*	*pseudoF*	R^2	*P*
RB1Y12 vs. RB1Y16	1	0.084	0.842	2.885	0.292	0.007
RB1Y12 vs. RB9Y16	1	0.066	0.066	2.184	0.238	0.019
RB9Y13 vs. RB1Y16	1	0.091	0.091	2.964	0.270	0.004
RB1Y12 vs. RB9Y13	1	0.036	0.036	1.268	0.153	0.084
RB9Y13 vs. RB9Y16	1	0.047	0.047	1.488	0.157	0.059
RB9Y16 vs. RB1Y16	1	0.068	0.068	2.109	0.209	0.032

Abbreviations: MS, mean sum of squares; SS, sum of squares
